# Dr. John W. Fawdry

**Published:** 1916-02

**Authors:** 


**Dr. John W. Fawdry** of Corfu died Nov. 10. Only one other physician is listed in the State Directory for Corfu—Dr. Joel Bates, a graduate of the Eclectic College of Cincinnati, 1875.

